# Effects of Different Numbers of Stimulations in Single-Session Radial Extracorporeal Shock Wave Therapy on Spasticity Improvement in Individuals With Subacute Hemiparetic Stroke: Protocol for a Pilot Randomized Controlled Trial

**DOI:** 10.2196/88443

**Published:** 2026-09-24

**Authors:** Daisuke Kato, Satoshi Hirano, Shota Itoh, Sora Ozeki, Toshiki Ito, Takuma Ishihara, Yohei Otaka

**Affiliations:** 1 Department of Rehabilitation Fujita Health University Hospital Toyoake, Aichi Japan; 2 Department of Rehabilitation Medicine School of Medicine Fujita Health University Toyoake, Aichi Japan; 3 Innovative and Clinical Research Promotion Center Gifu University Hospital Gifu, Gifu Japan

**Keywords:** muscle spasticity, physical therapy modalities, pilot projects, range of motion, rehabilitation

## Abstract

**Background:**

Radial extracorporeal shock wave therapy (rESWT) improves lower-limb spasticity after stroke. However, the efficacy of rESWT in individuals with hemiparetic stroke in the subacute phase, in whom neural components of spasticity are thought to predominate, has not been sufficiently investigated. In addition, the effects of different numbers of stimulations on the magnitude and duration of spasticity improvement remain unclear.

**Objective:**

We aim to explore the effects of different numbers of stimulations during a single rESWT session on lower-limb spasticity in individuals with subacute stroke and assess the feasibility and safety of the evaluation and treatment procedures for future large-scale trials.

**Methods:**

This single-center, open-label pilot randomized controlled trial will enroll 24 individuals with subacute stroke and spasticity who will be randomly assigned to either the rESWT 3000- or 6000-stimulation group for 1 treatment at the triceps surae muscle, with stimulation applied at 3 locations: the medial and lateral heads of the gastrocnemius muscle and the triceps surae muscle–Achilles tendon junction. The stimulation intensity will be the maximum intensity tolerated by the individual (≤3.0 bar), with a 10-Hz fixed frequency. Evaluations will be performed the day before treatment; immediately before and after completion of treatment; at 1 and 8 hours after treatment; and at 1, 2, 3, 5, 7, and 14 days after treatment. The primary clinical outcome is the intergroup difference in the Modified Ashworth Scale score of the triceps surae immediately after completion of treatment. Secondary clinical outcomes include intergroup differences in Modified Ashworth Scale scores at 1 and 8 hours and on days 1, 2, 3, 5, 7, and 14 after treatment, as well as intragroup differences from baseline to each evaluation point. Additional measures include the range of motion, angle of catch, tendon reflexes, and clonus scores. Feasibility will be assessed using the recruitment, enrollment, adherence, and retention rates. Safety will be assessed by recording the occurrence of serious and treatment-related adverse events.

**Results:**

This study was approved by the Fujita Health University Institutional Review Board on September 26, 2024 (CR24-033). Participant enrollment commenced on November 19, 2024. By November 25, 2025, a total of 14 participants had been enrolled. Data analysis has not commenced, and the results will be reported in a separate article in 2026.

**Conclusions:**

This exploratory dose comparison pilot study is designed to explore whether the number of stimulations delivered during a single rESWT session influences the extent of short-term improvement in spasticity following subacute hemiparetic stroke and obtain preliminary clinical outcomes and feasibility and safety data for future large-scale trials.

**Trial Registration:**

Japan Registry of Clinical Trials jRCTs042240098; https://jrct.mhlw.go.jp/en-latest-detail/jRCTs042240098

**International Registered Report Identifier (IRRID):**

DERR1-10.2196/88443

## Introduction

The management of spasticity is an important issue for individuals with hemiparetic stroke. Spasticity—a positive symptom of upper motor neuron disorders—occurs in 4% to 43% of survivors of stroke [1] and causes functional impairments such as joint contractures and pain and a decrease in activities of daily living and quality of life [2]. Therefore, the treatment and management of spasticity in survivors of stroke are important.

Radial extracorporeal shock wave therapy (rESWT) can effectively improve poststroke spasticity. The poststroke recovery process is generally categorized into several phases: the acute phase (1-7 days after stroke onset), the subacute phase (7 days to 6 months after onset), and the chronic phase (>6 months after onset) [3]. A systematic review and meta-analysis demonstrated that rESWT induces immediate improvement in lower-limb spasticity among individuals with chronic stroke, with the treatment-related effects lasting for more than 1 month [4]. However, only a few reports have detailed spasticity in the subacute phase after a stroke. The subacute phase is characterized by increasing spasticity, with some reports indicating a peak at 1 to 3 months after stroke onset [5,6]. Spasticity is a complex phenomenon that involves neural and nonneural components [7], and its composition possibly changes over time after a stroke [8-10]. The neural component, which is characterized by increased excitability of the stretch reflex, peaks during the subacute phase at 1 to 3 months after stroke onset [9]. In contrast, in the chronic phase, the contribution of nonneural components such as muscle and connective tissue increases [10]. These stage-specific pathophysiological changes may affect the therapeutic effects [11]. Although rESWT is thought to improve spasticity through both neural and nonneural mechanisms, the relative contribution of each remains unclear [12-16], particularly during the subacute phase. Therefore, it is essential to investigate the effects of rESWT on spasticity during the subacute phase after a stroke.

Additionally, an optimal treatment protocol has not yet been established, warranting investigation [17]. In extracorporeal shock wave therapy, the device type (radial or focused), stimulation intensity, stimulation site, and number of stimulations can be selected; these factors potentially influence the therapeutic effect. Regarding the device type, the radial type is more effective than the focused type [18]. Stimulation of both the muscle belly and the muscle-tendon transitional region is effective, and combined application is recommended for greater efficacy [19]. Furthermore, higher-intensity stimulation is more effective [20]. However, it remains unclear whether different numbers of stimulations influence the magnitude or duration of spasticity improvement during or after rESWT and whether such differences are associated with neural or nonneural components of spasticity. The number of stimulations has not been directly compared in randomized controlled trials (RCTs) examining rESWT effects. A systematic review and meta-analysis of studies conducted primarily in individuals with chronic stroke showed no significant difference in spasticity improvement between 1500 and 4500 stimulations per treatment session [21], but a recent RCT using focused extracorporeal shock wave therapy in individuals with chronic stroke suggested a dose-response relationship, with greater improvements following a higher number of stimulations [22]. Because previous studies have been heterogeneous regarding patient characteristics, extracorporeal shock wave therapy devices, and treatment protocols, the effects of the number of rESWT stimulations on treatment outcomes remain to be investigated in individuals with subacute stroke.

In this exploratory dose comparison pilot study, we aim to explore whether different numbers of stimulations in single-session rESWT influence the magnitude and duration of spasticity improvement in individuals with hemiparetic stroke in the subacute phase and generate preliminary clinical outcomes and feasibility and safety data to inform the design of future large-scale clinical trials.

## Methods

### Study Design and Setting

This study is designed as a single-center, parallel-group exploratory dose comparison RCT with a 1:1 allocation and is reported in accordance with the SPIRIT (Standard Protocol Items: Recommendations for Interventional Trials) 2013 statement [23], which was the latest version available at the time of protocol development. The protocol content has been summarized in accordance with the SPIRIT 2025 guidelines, and a completed SPIRIT 2025 checklist can be found in Multimedia Appendix 1. The results of this pilot trial will be reported in accordance with the CONSORT (Consolidated Standards of Reporting Trials) 2010 statement extension for randomized pilot and feasibility trials [24]. The study flowchart is shown in Figure 1. Survivors of stroke will be randomly assigned to either the rESWT 3000- or 6000-stimulation group. This study will be conducted at Fujita Health University Hospital (Aichi, Japan). As this protocol includes the statistical analysis plan, no separate statistical analysis plan has been created. The trial registry provides public access to the protocol, including the statistical analysis plan.

**Figure 1:**
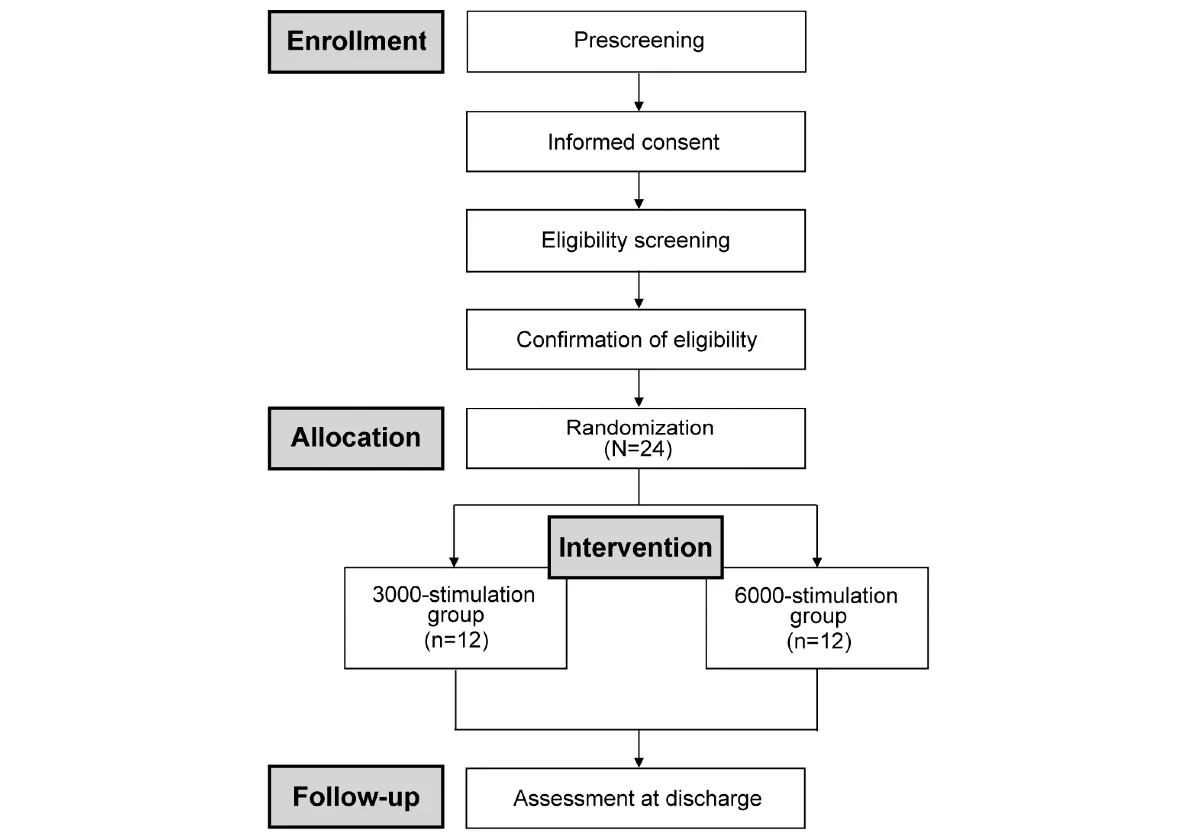
Flow diagram of the study process.

### Participants

The inclusion criteria are as follows: (1) provision of written informed consent by the participant or a legal representative, (2) age of 18 years or above and below 90 years, (3) presence of hemiparesis, and (4) a Modified Ashworth Scale (MAS) score of 1+ or higher in the triceps surae muscle.

The exclusion criteria are as follows: (1) damage to the skin at the treatment site; (2) acute inflammation; (3) thrombosis; (4) current or possible pregnancy; (5) tumors; (6) multiple neuropathies; (7) use of anticoagulants; (8) history of orthopedic disease at the treatment site; (9) pain during passive movement at the treatment site; (10) botulinum toxin therapy within the previous 4 months; and (11) any other condition that is deemed unsuitable by the attending physician, principal investigator, associate investigator, or research assistant.

### Recruitment

The participants will be identified from among patients who are hospitalized in the intensive rehabilitation ward of Fujita Health University Hospital. Preliminary screening will be conducted by 4 physical therapists with at least 5 years of experience, and board-certified rehabilitation physicians will carefully determine eligibility based on the screening results.

### Randomization

Participants will be randomly assigned to either the rESWT 3000- or 6000-stimulation group using a computer-generated permutation block randomization method with block sizes of 2 or 4. The randomization sequence will be generated by an independent data manager and incorporated into REDCap (Vanderbilt University) [25]. Investigators responsible for enrollment will register participants and press the allocation button on REDCap, at which point the assignment will be automatically displayed.

### Interventions

Participants will receive a single rESWT session during hospitalization. Treatment will be delivered using the BTL-6000 Topline (BTL Japan). As the intervention is provided during hospitalization, compliance will be ensured by performing the procedure on the scheduled day. The treatment site will be the triceps surae muscle, with stimulation applied to 3 locations: the medial and lateral heads of the gastrocnemius muscle and the muscle-tendon transitional region between the triceps surae muscle and the Achilles tendon. The stimulation intensity will be set to the maximum that is tolerated by the individual (upper limit: 3.0 bar), the frequency will be fixed at 10 Hz, and the number of stimulations will be set to 3000 or 6000 depending on the assigned group. Previous studies have used approximately 1500 to 4500 stimulations per treatment session [21]. We selected the 3000-stimulation protocol as the reference condition because it represents the midpoint of this commonly used range. The 6000-stimulation protocol was subsequently defined as a higher-dose exploratory condition by doubling the number of stimulations used in the reference protocol, allowing for the investigation of potential dose-related differences while maintaining clinical feasibility within a single treatment session.

From the day before treatment until the 14-day posttreatment time point, the use of the following medical devices and therapies will be prohibited: medical devices including electrical, magnetic, and vibration stimulation devices for the lower limbs; therapies including nerve block therapy; and changes in antispasmodic drugs (dosage or type).

The intervention will be discontinued if the participants withdraw, their condition deteriorates, they are found to not meet the eligibility criteria, or the investigators decide that the treatment should be stopped owing to adverse events such as severe pain or severe subcutaneous bleeding.

### Outcomes

#### Clinical Outcomes

The SPIRIT diagram in Figure 2 summarizes the data collection schedule. Evaluations will be conducted on the day before treatment; immediately before and after completion of treatment; 1 and 8 hours after treatment; and 1, 2, 3, 5, 7, and 14 days after treatment. The assessment on the day before will be used to confirm eligibility, whereas the assessment immediately before treatment will serve as the baseline for the primary analysis. Evaluations will be conducted by 1 of 4 physical therapists, each with at least 5 years of experience, who are different from the treatment implementers. The evaluators will confirm the evaluation methods before starting the study. The primary clinical end point will be the intergroup difference in immediate posttreatment MAS scores of the triceps surae muscle. The secondary outcomes will be the intergroup differences in MAS scores of the triceps surae muscle at 1 and 8 hours and 1, 2, 3, 5, 7, and 14 days after treatment, as well as the intragroup changes from baseline to each assessment point. Additionally, intergroup differences and intragroup changes in the angle of catch (AOC), range of motion (ROM), tendon reflexes, and clonus scores of ankle dorsiflexion will be assessed at the same time points.

The MAS is a reliable and valid indicator of muscle tone in survivors of stroke [26,27]. It is evaluated on a 6-point scale ranging from 0 (no increase in muscle tone) to 4 (indicating rigidity). For statistical analyses, the original MAS categories of 0, 1, 1+, 2, 3, and 4 will be numerically coded as 0, 1, 2, 3, 4, and 5, respectively. The MAS evaluation will be performed with the knee in an extended position. The ROM and AOC during ankle dorsiflexion will be measured using an electronic goniometer (Biometrics Ltd) at a sampling frequency of 1000 Hz. With the participant sitting on a chair with a backrest, the knee joint will be extended, and the ROM and AOC of the ankle joint dorsiflexion will be measured. The evaluator will move the participant’s joints as follows: (1) dorsiflex the ankle joint slowly to its maximum ROM, (2) plantar flex the ankle joint to its maximum ROM, and (3) dorsiflex the ankle joint rapidly to its maximum ROM. The evaluator will record the maximum ROM during procedure 1 and the ROM at the point of catch during procedure 3 as the AOC. A catch is defined as the point at which the acceleration in the plantar flexion direction of the ankle joint reaches its maximum [28]. The measurement will be performed twice, and the average value of the 2 measurements will be used for analysis.

The clonus score is a 5-point scale for clonus caused by upper motor neuron dysfunction based on the clonus duration (0=no clonus; 1=clonus for 1-4 seconds; 2=clonus for 5-9 seconds; 3=clonus for 9-15 seconds; and 4=clonus for >15 seconds [29]). The clonus score will be assessed with the knee joint in extension, with the patient seated in a chair with a backrest. The Achilles tendon reflex will be assessed based on the British scale for reflex grading, with 0 indicating reflex absent; 1+ indicating small, less than normal reflex, including a trace response or a response brought out only with reinforcement; 2+ indicating brisk reflex within the median normal range; 3+ indicating enhanced reflex, including high normal or hyperreflexia; 4+ indicating enhanced reflex, more than normal, including intermittent clonus; and 5+ indicating sustained clonus [30]. This reflex will be assessed with the knee joint in flexion, with the patient seated in a chair with a backrest.

Additionally, to characterize the participants, the following data will be collected at baseline and discharge: age, sex, height, weight, type of stroke, days from stroke onset to intervention, affected side, medication status, Glasgow Coma Scale (GCS) score, Stroke Impairment Assessment Set (SIAS) motor and sensory function scores for the lower extremities, Functional Ambulation Categories (FAC), and Functional Independence Measure (FIM) transfer and locomotion.

The GCS consists of 3 items: eye opening (scored as 1-4), verbal responses (1-5), and motor responses (1-6). The total GCS score, ranging from 3 to 15 points, is the sum of the scores of these 3 items, with 3 indicating severe impairment and 15 reflecting normal responsiveness [31]. The FAC is a reliable and validated clinical measure of gait ability based on the level of assistance required [32,33]. The scale consists of 6 levels, from 0 (unable to walk) to 5 (independent walking, including advanced walking activities). The SIAS serves as a reliable and validated index for assessing functional impairment in survivors of stroke [34-36]. It accounts for motor function, tension, sensory function, ROM, pain, trunk function, higher brain function, and muscle strength on the nonparalyzed side, with total scores of 0 to 72 points. The SIAS lower-limb motor function items consist of 3 tests, hip flexion, knee extension, and ankle dorsiflexion and are evaluated on a 6-point scale (score range 0-5 points). The lower-limb sensory function items include 2 tests: superficial sensation (touch and pain) and proprioception (joint position sense), wherein each is evaluated on a 4-point scale from 0 to 3. The FIM, comprising 13 motor items and 5 cognitive items, is a reliable index for assessing independence in activities of daily living [37,38]. Each item is rated on a scale from 1 to 7, with scores of 1 to 4 points indicating the need for varying levels of physical assistance, 5 points indicating the need for supervision, 6 points indicating modified independence, and 7 points indicating complete independence.

**Figure 2:**
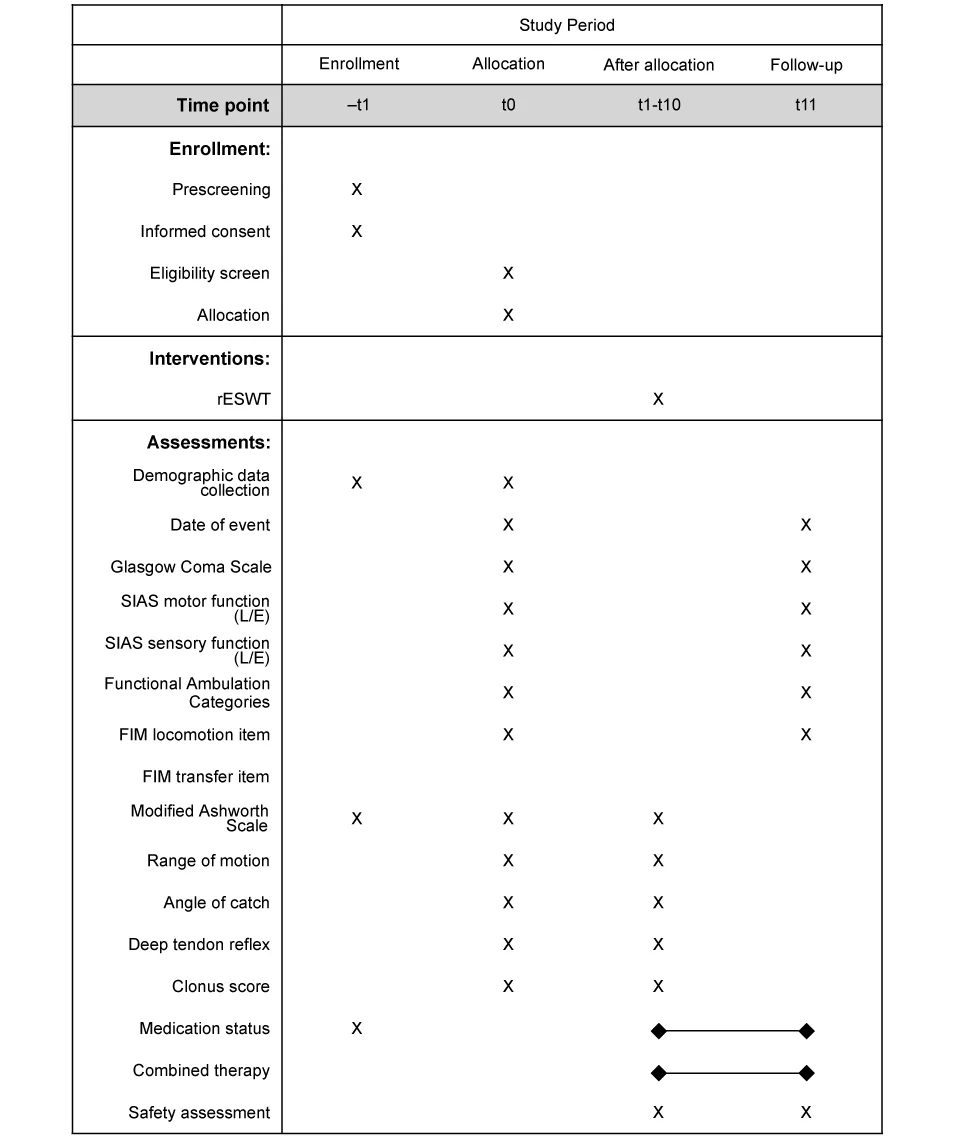
SPIRIT (Standard Protocol Items: Recommendations for Interventional Trials) diagram illustrating the enrollment, intervention, and assessment schedule according to the 2013 SPIRIT guidelines. FIM: Functional Independence Measure; L/E: lower extremity; SIAS: Stroke Impairment Assessment Set; −t1: preallocation assessment; t0: day before a single session of radial extracorporeal shock wave therapy (rESWT); t1: immediately before treatment; t2: immediately after completion of treatment; t3: 1 hour after the rESWT session; t4: 8 hours after the rESWT session; t5: 1 day after the rESWT session; t6: 2 days after the rESWT session; t7: 3 days after the rESWT session; t8: 5 days after the rESWT session; t9: 7 days after the rESWT session; t10: 14 days after the rESWT session; t11: follow-up assessment before discharge.

#### Feasibility Assessment

In accordance with previous recommendations [39], the feasibility of this pilot study will be evaluated based on the following indicators: (1) recruitment rate (average number of participants enrolled per month), (2) enrollment rate (the proportion of individuals who consent to participate among eligible individuals), (3) adherence rate (the proportion of participants who complete the allocated intervention as planned among all enrolled participants), and (4) retention rate (the proportion of participants who complete the outcome assessments among enrolled participants).

These measures will serve as progression criteria to determine whether to proceed with the study [40]. The specific criteria are as follows: (1) recruitment rate of at least 1.0 participants per month, (2) enrollment rate of at least 90% of screened eligible individuals consenting to participate, (3) adherence rate of at least 90% of participants completing the planned intervention, and (4) retention rate of a minimum of 90% of enrolled participants completing all outcome measures up to the final follow-up assessment. Recruitment rate criteria were established based on our institution’s experience, whereas the enrollment, adherence, and retention rates were determined with reference to previous pilot and feasibility studies in rehabilitation research [41]. Even if one or more of the 4 criteria are not met, the study will proceed with protocol modifications when the underlying issues are considered addressable [40].

#### Safety Assessment

The safety of the treatment will be evaluated by monitoring for serious adverse events and treatment-related adverse events (eg, subcutaneous hemorrhage, redness, and pain). Serious adverse events will be identified based on the guidelines for the occurrence of 5 events (death, life-threatening condition, hospitalization or prolonged hospitalization, persistent or significant disability or incapacity, and congenital anomaly or birth defect) [42]. Upon confirmation of a serious adverse event, the principal investigator will promptly report it to the institutional review board and the Ministry of Health, Labor, and Welfare. Adverse events related to the treatment, such as subcutaneous hemorrhage, redness, and pain, will be recorded. In addition to the immediate posttreatment assessment, the patient’s condition will be re-evaluated the day after treatment to confirm whether the event persists. If the event persists, it will be classified as an adverse event, and the responsible therapist will prepare the adverse event report. The adverse event report will include the name of the adverse event, date of incidence, details of its course, treatment provided, and subsequent outcomes. A report will be generated for each serious or treatment-related adverse event and compiled at participant discharge.

### Data Management

Research data will be entered and managed using REDCap, hosted by Fujita Health University [25]. Identifiable personal information will be deleted, and only coded and anonymized data will be used. All data will be password protected, with access restricted to authorized research personnel. Data quality will be ensured through predefined range checks and routine monitoring by the research team. In addition, independent trial monitoring will be conducted by an external observer not involved in this study prior to, during, and after the study to ensure protocol adherence and data quality. Access to the final trial dataset will be restricted to the principal investigator and authorized members of the research team.

### Sample Size

As this is a pilot study, an effect size–based sample size calculation was not performed based on statistical testing power [24] because of the limited prior data available for individuals with hemiparetic stroke in the subacute phase receiving equivalent rESWT protocols. We applied the recommendation of the “rule of thumb,” which suggests a minimum of 12 participants in each group [43]. This sample size was also considered feasible for this exploratory pilot study and should be sufficient to estimate means and variances and provide data on clinical outcomes.

### Blinding

There will be no blinding of participants, researchers, or outcome assessors because of the open-label study design. As all the parties are aware of the group allocation, there are no conditions for the nonblinding of information.

### Analytical Methods

The feasibility results will be reported as descriptive statistics. The recruitment rate will be calculated by dividing the number of participants enrolled by the number of months from the start to the end of the trial. The enrollment rate will be calculated as the number of participants who agree to take part divided by the number of eligible participants multiplied by 100. The adherence rate will be calculated as the number of participants who complete the allocated intervention as planned divided by the number of enrolled participants multiplied by 100. The retention rate will be calculated as the number of enrolled participants who complete all outcome measures up to the final follow-up assessment divided by the number of enrolled participants multiplied by 100. The recruitment, enrollment, adherence, and retention rates will be reported with 95% CIs. Adverse events and their frequencies will be recorded. The reasons for and the time points of potential exclusions or dropouts will be recorded.

For the primary outcome analysis, the mean and SD of the MAS score of the triceps surae muscle after completion of treatment will be calculated for each group, as well as the mean and SD of the between-group difference. For the secondary outcomes, the mean and SD of the MAS score of the triceps surae muscle, ankle dorsiflexion AOC, ROM, tendon reflexes of the triceps surae, and clonus score will be calculated at each assessment time point for each group, along with the mean and SD of the between-group differences. For each group, the means and SDs of the changes from immediately before treatment to each posttreatment assessment time point will also be calculated for the MAS of the triceps surae, ankle dorsiflexion AOC, ROM, tendon reflexes of the triceps surae, and clonus score. Although the MAS score, tendon reflexes, and clonus score are ordinal clinical scales, these measures have frequently been numerically coded and summarized using means and SDs in previous spasticity studies [44-47]. Accordingly, these outcomes will be summarized descriptively using means and SDs in this exploratory pilot study. Furthermore, a mixed-effects model will be used with the MAS score of the triceps surae as the dependent variable to examine the between-group differences at each time point. Fixed effects will include the MAS score immediately before treatment as a covariate, group, time, and the group × time interaction, with participant as a random effect. The variance-covariance structure will be specified as a first-order autoregressive covariance structure; if the model fails to converge, compound symmetry will be applied. Least squares means will be estimated at each time point. The primary contrast will be the between-group difference in the MAS score immediately after completion of treatment. Model-based estimated between-group differences and corresponding 95% CIs will be calculated and reported; the between-group differences will be tested under the null hypothesis that the difference equals zero. The within-group changes in secondary outcomes will be descriptively summarized to characterize the time course and persistence of treatment-related changes rather than formally tested inferentially in this exploratory pilot study. A 2-sided significance level of 5% will be used for all analyses. Adjustment for multiple comparisons will not be performed because these analyses are exploratory and not intended for confirmatory hypothesis testing. The statistical hypothesis tests are intended to provide preliminary estimates of treatment-related changes and their variability for future trials. The risk of missing data is considered to be low because all evaluations are conducted during hospitalization. For sensitivity analyses, the missing values will be imputed using the last observation carried forward method to assess the robustness of the findings.

### Patient and Public Involvement

Patients or the public were not involved in the design, conduct, reporting, or dissemination plans of this research.

### Ethical Considerations

This study protocol was approved by the Fujita Health University Institutional Review Board on September 26, 2024 (approval CR24-033) and was registered in the Japan Registry of Clinical Trials on October 4, 2024 (jRCTs042240098). In accordance with the principles outlined in the Declaration of Helsinki of 1964 (revised in 2013), written informed consent will be obtained from all participants (or their family members or legal representatives if the patients are unable to consent) prior to participation. The findings will be disseminated through peer-reviewed publications and conference presentations. Additionally, a summary of the key findings will be made available on the trial registry’s website, and upon request, participants and their families will receive an intelligible summary of the findings.

## Results

Participant enrollment commenced on November 19, 2024. By November 25, 2025, a total of 14 participants had been enrolled. Enrollment is expected to be completed by September 30, 2026. Data collection commenced in November 2024 and is expected to be completed in October 2026. Data analysis has not commenced, and the study’s results will be reported in 2026.

## Discussion

This exploratory pilot study is expected to provide preliminary insights into whether differences in the number of rESWT stimulations influence the magnitude and duration of spasticity improvement in individuals with hemiparetic stroke in the subacute phase and obtain clinical outcomes and feasibility and safety data that will inform the design of future large-scale clinical trials. We hypothesize that the antispastic effects of rESWT in the subacute phase may attenuate more rapidly than those observed in previous studies involving patient populations with chronic stroke, thereby requiring frequent short-term assessments to appropriately characterize the temporal profile of treatment effects. We also hypothesize that a higher number of stimulations may increase the magnitude or duration of short-term spasticity improvement.

Poststroke spasticity varies over time in terms of both severity and the underlying neural and nonneural components [5-10]. Neural components are considered to predominate in the subacute phase [9], whereas nonneural components are thought to be dominant in the chronic phase [10]. Although rESWT is effective in alleviating poststroke spasticity, most previous studies have focused on patients in the chronic phase [4]. Therefore, evidence obtained from chronic-phase populations may not directly apply to subacute-phase individuals, whose underlying spasticity components differ. Thus, investigating rESWT in populations with subacute stroke, as well as conducting large-scale trials, is considered to be of both clinical and scientific importance. In fact, we previously encountered an individual with hemiparetic stroke in the subacute phase in whom the rESWT effects persisted only for several days, which motivated the development of this pilot study [48]. If the rESWT effects indeed are attenuated within a few days, conventional weekly or monthly assessments may fail to accurately capture the temporal profile of treatment effects. Therefore, we designed a novel assessment protocol involving short-term and frequent assessments.

Regarding the effects of the number of stimulations on treatment outcomes, previous meta-analyses have not demonstrated a clear dose-response relationship [21]. However, these findings were not based on direct comparisons within a single study, and heterogeneity in other treatment parameters across studies may have obscured a potential dose-response relationship. In contrast, a prior study using focused extracorporeal shock wave therapy suggested the presence of a dose-response effect, indicating that a similar relationship may also exist in rESWT [22]. An increase in the number of stimulations may be associated with longer treatment duration and greater patient burden. Therefore, in anticipation of future large-scale trials, it is important for this study to provide preliminary insights into determining an appropriate number of stimulations. If the number of stimulations does influence treatment outcomes, future large-scale studies should consider adopting the stimulation parameters that yield the greatest therapeutic benefit.

This study has several limitations. First, this protocol describes an open-label trial. The lack of assessor blinding might influence the results. Although we considered conducting blinded assessments by independent evaluators, constructing an operational framework capable of carrying out frequent assessments over a short period using resources within a single rehabilitation center proved difficult. As the primary outcome, the MAS score is susceptible to assessor bias; therefore, measures were taken to reduce interrater variability by restricting assessments to 4 physical therapists with extensive experience in stroke rehabilitation and providing them with prior training on the assessment procedure. However, the possibility of residual bias cannot be excluded. Therefore, future large-scale trials should consider research designs that allow for the blinding of outcome assessments. Second, this study lacks a sham-treated or non-rESWT control group. Although comparisons between the 3000- and 6000-stimulation groups may provide preliminary information regarding dose-related differences, it may be difficult to determine the extent to which any observed changes are attributable to rESWT itself rather than natural recovery or spontaneous fluctuations in spasticity during the subacute phase after stroke. Therefore, future definitive trials should consider including an appropriate sham-controlled design to better isolate the treatment effects of rESWT. Third, this is a small-scale, single-center study. Therefore, the findings should not be interpreted as evidence of the efficacy of rESWT but rather as preliminary results intended to inform the design of future large-scale trials. Fourth, no restrictions were placed on usual rehabilitation during the study period, which might influence spasticity outcomes. However, the contents of rehabilitation will be recorded, and potential imbalances between groups will be assessed and considered when interpreting the results. Fifth, the analysis plan for this study involves treating the MAS score as a continuous variable and applying a mixed-effects model, although the MAS is an ordinal scale. This method may not fully satisfy the assumptions of linear modeling. In this small exploratory pilot study, ordinal regression models may not provide stable estimates. However, similar approaches have been applied in previous longitudinal studies [49,50]. Future large-scale studies should carefully consider the most appropriate statistical model for MAS outcomes.

To our knowledge, this is the first study to evaluate the effect of the number of rESWT stimulations on lower-limb spasticity in individuals with hemiparetic stroke in the subacute phase. The findings of this exploratory pilot study are expected to provide preliminary clinical outcomes and feasibility and safety data that will inform the design of future large-scale RCTs.

## Appendix

Multimedia Appendix 1 SPIRIT (Standard Protocol Items: Recommendations for Interventional Trials) checklist.

## Abbreviations

AOC: angle of catch
CONSORT: Consolidated Standards of Reporting Trials
FAC: Functional Ambulation Categories
FIM: Functional Independence Measure
GCS: Glasgow Coma Scale
MAS: Modified Ashworth Scale
RCT: randomized controlled trial
rESWT: radial extracorporeal shock wave therapy
ROM: range of motion
SIAS: Stroke Impairment Assessment Set
SPIRIT: Standard Protocol Items: Recommendations for Interventional Trials

## Data Availability

Deidentified individual participant data, the data dictionary, and the analysis code will be made available on reasonable request to the corresponding author after the publication of the trial results. However, no identifiable information will be shared.
